# Remarks on the History and Use of Tobacco

**Published:** 1811-02

**Authors:** 


### *Remarks on the History and Use of Tobacco.*

(Continued from vol. xxiv. p. 460.)

THE plant which we have seen (*Med. and Phys. Journal*, xxiv. p. 445) disturbing, for a century or more, the repose of the statesman, the priest, and the moralist; and awakening the poet's lyre, to censure or to praise; is an individual of a genus containing seven species.* In the Linnæan sys-

* The six individuals of this family, which differ from the *Nicotiana tabacum* in external appearance, (and probably in physical properties), of sufficient permanence to constitute species, are,

1st. NICOTIANA *fruticosa*. Shrubby tobacco. (Lin. S. 258. Reich I. 502. Lour. Cochinchina, 111. Burm. Ind. 54. Rumphius Amboi. 7. L. 8. C. 40.) This is, perhaps, indigenous in China, Cochinchina, and India; and, possibly, like the Ginseng, may be found growing spontaneously in some particular spots both in the old and new world. Sir George Staunton informs us (Embassy 2, 174,) that it is planted in great quantities in the low grounds of China. On the continent of Hindoostan, and in the islands of the Indian ocean, it is also cultivated with much attention. In Java, particularly about Batavia, it grows to the heighth of eight feet, with leaves eighteen inches long and eight broad (Nieuhoff.) In Ceylon (Phil. Trans.) two varieties were cultivated. It grows naturally in the woods of the island of Tobago, tem this genus is placed in the class *pentandria*, order *monogynia*; and belongs, in the natural arrangement, to the

---

Tobago, from whence the seeds were sent to Phillip Miller. (Martyn.)

Var. *N. alba*. Mill. Dict. No. 5.

—— *N. major latifolia*, floribus albis, vasculo brevi. Martyn, Hist. Plant. rarior. cent. 5.

(2) Nicotiana *rustica*, common or English Tobacco. (Lin. spec. 258. Reich. 1. 503. Hort. Cliff. 56-2. Ups, 45. Blackw. t. 437. Kniph. cent. 3. n. 65. Sabb. hort. 1. t. 90. Berg. phyt. 2. 57. Plenck, icon. 100.)

The *N. rustica* was the species first imported, and being more hardy than its congenors, is become a weed in many parts of England. It came originally from America by the name of *Petume*, a Brazilian word. This species is also rendered interesting from being that which Sir W. Raleigh smoked during his confinement in the Tower; and which he cured himself, says Parkinson his contemporary, with great nicety. Sir W. Raleigh, on his return from America, is said to have introduced the smoking of Tobacco into England. In the house wherein he lived at Islington, now the Pied Bull public-house, his arms remain, with a Tobacco plant on the top of the shield. In this house perhaps it was, when soothing his care-worn mind by indulging in his favourite relaxation, and

> Faucibus ingentem fumum, mirabile dictu,

> Evomit; involvitque domum caligine cœca,

> Prospectum eripiens oculis; glomeratque sub antro

> Fumiferam noctem, commistis igne tenebris.

>

> Virgil.

that his honest English servant, supposing Sir Walter to be on fire, deluged him with water. It may be a question how far the fate of this valuable man was influenced by the notion of his having taught the English to smoke. James had strong prejudices, and his irritations on the subject of Tobacco might have been transferred from the plant to the man. He believed the morals, and even the natural powers of his people to be depraved and diminished by the use of Tobacco. To destroy the man who imported the infernal practice might appear to his mind an act of justice.

*Synon. N. minor*. Morison, hist. 493. 4. f. 4. *Tabaccum anglicum*. Park. theat. 711. t. 712. *Hyoscyamus luteus*. Dod. pempt. 450. Ger. 284. emac. 356. *Priapeia quibusdam Nicotiana minor*. Bauh. hist. 3. 630. f. 3. Raii hist. 715. *Pachyphylla*. Renealm. spec. 40.

Var. *N. rugosa*. Mill. dict. n. 7.

—— *N. minor*, fol. rugosioribus amplioribus. Vaillant.

(3) Nicotiana *paniculata*. Panicled Tobacco. Linn. spec. 259. Reich. 1. 503. Kniph. cent. 2. n. 48. Feuill. Peruv. 1. 717. t. 10.

The *N. paniculata* is a native of Peru, was cultivated by Miller in 1739, who says it was found in the valley of Lima by Pere Feuillée in 1710, since which, the seeds were sent from Peru by the younger Jussieu to Paris. The stalk of the panicled Tobacco rises more than three feet, dividing upwards into many panicled branches, which are

LURIDÆ.* The denomination *Nicotiana* has been given to it, from the M. Nicot, before noticed.

---

round and somewhat hairy. Leaves about four inches long, and three broad, upon longish footstalks. Flowers in loose panicles at the ends of the branches: tube about an inch long and club shaped; brim slightly cut into nine obtuse reflex segments: corolla of a yellowish green, and succeeded by a roundish capsule.

(4) NICOTIANA *urens*. Stinging Tobacco.

Lin. spec. 259. Reich. 1. 503.

A native of South America. The fructification of this species is in racemes directed one way and revolute, with bell shaped corollas, and cordate leaves like those of N. rustica, but crenate, the stem hispid, stinging; and the whole tree prickly.

*Synon.* N. arborescens spinosissima, flore ex albido. Plum. spec. 3. icon. 211.

(5) NICOTIANA *glutinosa*. Clammy-leaved Tobacco. Lin. spec. 259. Reich. 1. 503. Kniph. cent. 10. n. 65.

The seeds of this were sent from Peru with the *N. urens* by the younger Jussieu, and cultivated by Millar in 1759. Stalk round near four feet high, sending out two or three branches from the lower part. Leaves large, heart shaped and a little waved, very clammy, standing upon long footstalks. Flowers in loose spikes (*racemes*) at the top of the stalk, of a dull purple colour; calyx unequally cut, one of the segments being twice the size of the others. The corolla almost ringent, gaping at the throat.

*Synon.* N. militaris. Act. holm. 1753. p. 41. t. 2.

(6) NICOTIANA *pusilla*.

Lin. spec. 258. Reich. 1. 504.

This species was discovered in La Vera Cruz, by Dr. Houston, who sent the seeds to England. It has a thick taper root, which strikes deep in the ground; at the top of it come out six or seven radical, oblong, oval leaves, about the size of those of the common primrose, but of a deeper green. The stalk rises about a foot high, branching into three or four divisions, at each of which is placed one small leaf. The branches are terminated by a loose spike of flowers, in acute racemes, which are small, and of a yellowish green colour.

The dealers in Tobacco have many varieties, which probably differ but little in their properties. In the time of *Jean Neander*, who published the first edit. of his Tabacalogia in 1622, there were known among the merchants the following: Varinium, Tecaporinum, Brasilianum, Ornochesium, Marcapanum, Craxium, Commenarinum, Gotegonasium, Burneodes, Amazonium, Manochesium, Comenagoticum, Virginiense, ex Insul. S. Margaritæ, ex Insul. Phillipinis, ex Insul. S. Lucæ, ex Insul. S. Trinitatis, ex Insul. S. Dominice. These seem to be arranged according to the order in which they were esteemed; the *Varinium* was considered as

* In the former part of these Remarks, Cynoglossum, Conium, and some species of Agarics are apparently referred to the natural order

The species, which is the subject of these Remarks, is the *Nicotiana Tabacum. Virginian Tobacco.**

It is described as having a large, long, fibrous, annual root:—an erect, strong, round, hairy stalk, branched to-

---

as the best. At present (1811) the London Tobacconists have K'naster two sorts, Virginia three sorts, Oroonoco (Maryland), Ellsham, Persian, Turkey, and Segars. The varieties of snuff are very numerous. They differ but little perhaps in their real properties; but when mixed with odoriferous substances, or changed in their appearance, they have the names of Princes, Dukes, and Generals given to them, and also of noted manufacturers.

*Luridæ.*† They are not, however, to be placed in that order. The Cynoglossum belongs to the Asperipoliæ; and the Conium to the Umbellatæ. Among that class of cryptogamic plants known under the denomination *Agaricus*, will be found some individuals having properties extremely deleterious to animal life. Many instances of their poisonous effects are on record; the pages of the Medical Journal will afford several: but there is not yet, to my recollection, a single notice of their having been employed medicinally. They have a strong action on the brain and nerves, exciting in these parts of the animal system extraordinary commotion; producing temporary insanity, and sometimes death. Three species, Agaricus muscarius, Agaricus glutinosus, and A. piperitidis are known, or suspected to possess the property of thus disturbing the functions: and one of them, the Agaricus muscarius, under the vernacular term *moucha-moura*, (so called by the Russians. Pennant Art. Zoo. 1. cxviii.) is employed in the northern parts of Europe and Asia for the same purpose that *Bangue* is used in India. Possessing these narcotic or stimulating powers in an uncommon degree, it is a question if they might not be employed medically under some circumstances. The extensive circulation of the Medical Journal may possibly occasion this hint to be improved into an experimental inquiry, that may elucidate this obscure and neglected subject.

* Lin. Spec. 258. Reich. 1. 502. Mat. Med. 64. Woodville Med. Bot. 162. t. 60. Hort. Cliff. 56. 1. Ups. 45. Blackw. t. 146. Kniphof Botanica in originali cent. 4. t. 55. Ludw. est. t. 167. Know. del. 1. t. 11. Sabb. Hort. 1. t. 89. Plenck icon. 99. Abbott georg. t. 33. Gærtn. fruct. 1. 264.

† Vid. *Fragmenta methodi naturalis* of Linnæus, which with the *canones*, or institutes, occupies 30 pp. of the *Classes Plantarum.* Edit. 8vo. Lugd. Batav. 1738. Linnæus, in his lectures, continued to add very considerably to the *Fragmenta methodi naturalis*; and as much as could be collected from these lectures, was afterwards published by Dr. Gieseke at Hamburg, under the title of *Caroli Linnæi Prælectiones in ordines naturales plantarum è proprio et fa. Chr. Fabricii, Prof.* Kilon. M Sto. Hamb. 1792. 8vo. pp. 662. cum tabb. n. 8, The nat. ord. *Luridæ*, the 33d in the *Fragmenta*, contains the following genera—Capsicum, Solanum, Physalis, Atropa, Hyoscyamus, Mandragora, Datura, Verbascum, Celsia, Digitalis, and Nicotiana.

wards the top, and rising five or six feet in height :—leaves numerous, large, oblong, pointed, entire, veined, viscid, pale green, without footstalks :—bractæ long, linear, pointed : —flowers in loose clusters, terminating the stem and branches :—calyx hairy, about half the length of the corolla, cut into five narrow segments :—corolla monopetalous, funnel shaped, tube hairy, gradually swelling toward the border, where it divides into five folding acute segments of a reddish colour :—filaments bent inward, tapering, and crowned with oblong anthera :—germen oval, supporting a long slender style, terminated by a round cleft stigma :—capsule ovate-conical, cloathed with the calyx, smooth, with four depressed streaks, two-celled, opening four ways at top :—partition simple, contrary to the valves :—receptacle very large, fungous, ovate acuminate, convex on one side, and flat on the other ; or reniform concave, fastened on both sides to the partition ;—seeds very numerous, small, ovate, subreniform, with raised lines or nerves beautifully reticulated, of a yellowish bay colour.*

Of this plant, which has been admitted into the *Materia Medica* of the London Pharmacopeia, there are several varieties,† all however of American growth, and possibly not

---

* Vide Gaertner, de Fructibus et Seminibus Plantarum, 2 vols. 4to. Stutgard, 1788, 1791. The exquisite plates of these volumes beautifully express the form and texture of fruits and seeds.

† Var. (1.) *N. latissima.* Mill. dict. n. 1. Oroonoco Tobacco. This is the sort commonly brought to market in pots : its leaves are more than a foot and a half long, and a foot broad, their surfaces very rough and glutinous, and their bases half embrace the stalk. In a rich moist soil the stalks are more than ten feet high, and the upper part divides into smaller branches, which are terminated by loose bunches of flowers, standing erect : they have pretty long tubes, and are of a pale purplish colour. Flowers in July and August, and its seed ripens in the autumn.

Var. (2.) *N. tabacum.* Mill. dict. n. 2. Broad-leaved Virginian, or sweet-scented Tobacco. *Synon.* (1) *N. major latifolia.* Bauh. pin. 169.—Mor. hist. 2. 492. f. 5. t. 11. f. 1. (2) *N. major.* Tabern. s. Tabacum majus. Bauh. hist. 3. 629. (3) *Tabacum latifolium.* Camer. and Besl. eyst. (4) *Tabacco latifolium.* Park. parad. 363. t. 361. f. 8. Raii hist. 713. (5) *Petum latifolium.* Class exot. 309. (6) *Sana sancta.* nob. adv 251. icon. 252. (7) *Hyoscyamus peruvianus.* Ger. 285. 1. emac. 357. 1. (8) *Tornobia.* Cæsalp. (9) *Blennochoes.* Renealm. spec. 37. t. 38. This variety flowers and perfects its seeds at the same time, and is sometimes called sweet-scented Tobacco. It is the broad-leaved Tobacco of C. Bauhin, seldom rises more than five or six feet, and divides into more branches than *Var.* 1. The leaves are about ten inches long, and three and a half broad, smooth, differing much in their properties. The *Nicotiana Tabacum* properly so called from being the most active and powerful of the genus, and distinguished in the preceding description from its congenors and its varieties, has been examined with minute precision as regards its operation on the mind and on the body—in its botanical, commercial, political, chemical, and medical history. In the former part of these remarks a detail was given of its discovery and first importation into the old world; its sudden and extensive spread among the European states; its influence upon the mind by its soothing and insidious qualities: and its agency in the delusive arts of the Pagan priesthood.* Having now also passed

---

smooth, acute, sessile: the flowers are rather larger than in *Var.* 1, and of a brighter purple colour.

VAR. (3.) *N. angustifolia.* Mill. dict. n. 3. fig. t. 185, f. 1. Narrow-leaved Virginian Tobacco.

*Syn.* (1.) *N. major angustifolium.* Bauh. pin. 169. Besl. eyst. Mor. hist. 429. 2. f. 2. (2.) *N. s. Tabacum fol. angustiora.* Bauh. hist. 3. 629. (3) *Tabacum angustifolium.* Camerar. (4) *Tabacum alterum minus.* Dalech. (5) *Tabacum s. herba sancta minor.* Lobel. adv. 251. (6) *Tobacco angustifolium.* Park. parad. 363. Raii. hist. 714. (7) *Petum angustifolium.* Class. exot. 309. (8) *Sanasancta Indorum.* Ger. 285. 2. emac. 357. 2. This rises with an upright branching stalk, four or five feet high. The lower leaves are a foot long, and three or four inches broad: those on the stalks are much narrower, lessening on the top, and end in very acute points, sitting close to the stalks; and are very glutinous. The flowers grow in loose bunches at the top of the stalks; they have long tubes, and are of a bright purple or red colour. They appear at the same time with *var.* 2, and ripen their seeds in the autumn. I am indebted for much information to the elaborate edition of Millar by Professor Martyn, who has so much improved the original work, that in justice it now should be called Martyn's rather than Miller's Dictionary.

* The natural simplicity, and the air of unaffected truth, with which the modes of incantation employed by the priests of the *aborigines* of America are described in an old translation of *Monardes*, B. L. 1580, may afford some apology for inserting the account here. "One of the mervelles of this hearbe (Tobacco) and that which bringeth most admiration, is, the maner howe the Priestes of the Indians did use it, which was in this manner: when there was amongst the Indians any manner of businesse, of great importance, in which the chief Gentlemen called *Casiques*, or any of the principall people of the countrie, had necessitie to consult with their Priestes, in any businesse of importance: then they went and propounded the matter to their chiefe Prieste, forthwith in their presence, he tooke certayne leaves of the Tobaco, and cast them into the fire, and did receive the smoke of them into his mouth, and at his nose with a cane, and in taking of it, hee fell downe upon the ground, through the scientific part of its botanical history, it remains to notice shortly its culture, and commercial importance; the discovery of its constituent materials by chemical analysis, and to point out its medical properties and its effects, whether curative or deleterious, on animal bodies.

In two points the cultivation of Tobacco has been an object of great solicitude to mankind. On its first introduction into Europe, the morning of science had scarcely broke on that long night of barbarian ignorance, which involved in intellectual darkness the greater portion of this division of the earth. The wonders told by travellers who had just returned from America, and especially of the properties of this plant, aided by its apparent effects, operated strongly on the feelings and expectations of the uninformed population of Europe. Tobacco was sought every where; and as soon as its seed could be procured, it was grown in every place.* When this inducement to its cultivation subsided, as it must when experience made it better known, another sprang up. Its promise to produce great wealth † both

---

ground, as a dead man, and remayning so, according to the quantitie of the smoke he had taken. When the hearbe had done his woorke, he did revive and awake, and gave them their answeares, according to his visions, and illusions which he saw, whiles hee was rapte in the same manner, and he did interprete them, as to him seemed best, or as the Divell had counselled him, giving them continually doubtfull answeares, in such sorte that howsoever it fell out, they might saye that it was the same, which was declared, and the answeare that he made."

"In like sort the reste of the Indians for their pastime doe take the smoke of the Tobaco, too make themselves drunke withall, and to see the visions, and things that represent unto them that wherein they doe delight: and other times they take it to know their businesse, and successe, because conformable to that, which they have seen beyng drunke therewith, even so they judge of their businesse. And as the Devill is a deceaver, and hathe the knowledge of the vertue of hearbes, so he did shew the vertue of this hearbe, that by the means thereof, they might see their imaginations and visions." Fol. 39.

* As all the species of *Nicotiana*, the *Rustica*, and *Paniculata* excepted, are too tender to be raised from seeds, without the assistance of artificial heat, it is probable the *N. Tabacum* was not generally grown in the fields and gardens of Europe. The *N. rustica* (*common or English Tobacco*) the favorite of Sir Walter Raleigh, most likely was the plant that invaded Europe, and threatened to dislodge the natural and useful possessors of the soil.

† The first commerce in Tobacco seems to have commenced about 1585, and as it proceeded, the demand for it in Europe became so urgent, that at the beginning of 1600, it was essential for the existence of the American colonies to restrain the cultivation, which by the hope to individuals and to states, gave it a second triumph: and the financier now became as anxious to promote its increase as the physician, who hailed it as an universal *panacea* had formerly been.

The culture of this plant as adopted in Europe, in a commercial view has now no importance. To science, however, it still affords no small portion of interest; and the philosophical botanist who desires to compare the species with each other, and fully to comprehend the generic characters, will not be displeased with the note* below, which describes from

---

hope of gain had extended so as to threaten the exclusion of grain of all kinds. For a detail of the progress of the culture and commerce of Tobacco from 1584 to 1748, Anderson's elaborate History of Commerce may be consulted; and to that excellent work I refer with confidence in its correctness.

* The seeds of the tender species of the Tobacco plant, viz. *N. tabacum*, *N. fruticosa*, *N. urens*, *N. glutinosa*, and *N. pusilla*, must be sown on a moderate hot-bed in March, and when the plants come up fit to remove, they should be transplanted into a new hot-bed of moderate warmth, about four inches asunder each way, observing to water and shade them until they have taken root; after which let them have air in proportion to the warmth of the season, otherwise they will draw up very weak, and be thereby less capable of enduring the open air: water them frequently, but while they are very young it should not be given to them in great quantities; though when they are pretty strong, they will require to have it often, and in plenty.

In this bed the plants should remain until the middle of May, by which time, if they have succeeded well, they will touch each other: therefore they should be inured to bear the air gradually; after which they must be taken up carefully, preserving a large ball of earth to each root, and planted into a rich light soil, in rows four feet asunder, and the plants three feet distant in the rows, observing to water them until they have taken root; after which they will require no further care (but only to keep them clean from weeds) until the plants begin to shew their flower-stems; at which time cut off the tops of them, that their leaves may be the better nourished, whereby they will be rendered larger, and of a thicker substance. In August they will be full grown, when they should be cut for use; for if they are permitted to stand longer, their under leaves will begin to decay. This is to be understood of such plants as are propagated for use, but those which are designed for ornament should be planted in the borders of the pleasure-garden, and permitted to grow their full height, when they will continue flowering from July till the frost puts a stop to them.

The *N. urens*, *glutinosa*, and *pusilla*, being somewhat more tender than the *N. tabacum*, when removed from the hot-bed in which they were sown, and when they have obtained a good share of strength, should be transplanted into separate pots, and plunged into a moderate hot-bed to bring them forward. About the middle of June some of the plants the best source, the judicious methods that the most rational gardeners of the present time employ. The growing of Tobacco in America* has the same interest which the cultiva-

---

plants may be shaken out of the pots, and planted in beds of rich earth; but it will be proper to keep one or two plants in pots, to be placed in the stove, in the case the season should prove bad, that they may ripen their seeds.

The *N. rustica*, and *paniculata* being very hardy, may be propagated by sowing their seeds in March upon a bed of light earth, whence they may be transplanted into any part of the garden, and will thrive without farther care. MARTYN's MILLER.

* In America Tobacco is cultivated with great care and attention; but the mode of management varies, as we see in England a different kind of agriculture pursued in the different counties. In 1724 the Rev. Hugh Jones, of James-town in Virginia, thus describes the culture of Tobacco. "When a tract of land is seated," says he, "they clear it by felling the trees about a yard from the ground, lest they should shoot again. What wood they have occasion for they carry off, and burn the rest, or let it lie and rot upon the ground.

"The land between the logs and stumps they hoe up, planting tobacco there in the spring, enclosing it with a slight fence of cleft rails. This will last for tobacco some years if the land be good, as it is where fine timber or grape vines grow.

"Land, when hired, is forced to bear tobacco by penning their cattle upon it; but cow-penned tobacco tastes strong, and that planted in wet marshy land is called *non-burning tobacco*, which smokes in the pipe like leather, unless it be of a good age.

"When land is tired of tobacco it will bear Indian corn or English wheat, or any other European grain or seed, with wonderful increase.

"Tobacco and India corn are planted in hills, as hops, and secured by *worm fences*, which are made of rails supporting one another very firmly in a particular manner.

"Tobacco requires a great deal of skill and trouble in the right management of it. They raise the plants in beds as we do cabbage plants, which they *transplant* and *replant* upon occasion after a shower of rain, which they call a *season*. When it is grown up they top it, or nip off the head, succour it, or cut off the ground leaves, weed it, hill it, and, when ripe, they cut it down about six or eight leaves on a stalk, which they carry into airy tobacco houses; after it is withered a little in the sun, there it is hung to dry on sticks, as paper at the paper mills; when it is in proper case (as they call it) and the air neither too moist, nor too dry, they *strike it*, or take it down, then cover it up in *bulk*, or a great heap, where it lies till they have leisure or occasion to *stem* it (that is, to pull the leaves from the stalk), or strip it (that is, to take out the great fibres), and tie it up in *hands*, or *straight lay it*, and so by degrees prize or press it with proper engines into great hogsheads, containing from about six to eleven hundred pounds; four of which hogsheads tion of wheat has in this country. It affords subsistence to multitudes. A failure in the crop of Tobacco is a national evil: because its produce gives revenue to the state, and wealth to the merchant; to the farmer competency, and to the labourer employment.

A circumstance which accompanies the cultivation of Tobacco in America, if the principle of it were not seen in Europe through the medium of similar facts, would be thought so wonderful as almost to forbid belief. This powerful soporific plant, which in a few grains only enervates the strongest man, and pushed a little further extinguishes the vital principle, is eaten with avidity, and is indeed the principal food of the *larva* of a delicate fly.*

---

heads make a tun, by dimensions, not by weight; then it is ready for sale or shipping.

"There are two sorts of tobacco, viz. *Oroonoco,* the stronger, and *sweet-scented,* the milder; the first with a sharper leaf like a fox's ear, and the other rounder and with finer fibres: but each of these are varied into several sorts, much as apples and pears are; and I have been informed by the Indian traders, that the inland Indians have sorts of tobacco much different from any planted or used by the Europeans." A German author describes, about 1794, the Virginian mode of culture and cure, which seems exercised with a ridiculous precision. In the voyages of *Pere le Bat* there are many particulars of the first methods of managing the tobacco plant; and in the American Museum for 1787 and 1789, an account will be found of the raising and curing of tobacco in Maryland, and of the method of cultivating that plant in Virginia where it borders on that country, as practised by Judge Parker. The great obstacles to the successful culture of tobacco in America, are the fly and the worm.

* It will be gratifying to me, and probably acceptable to most of the Readers of the Medical Journal, to have described by some of its numerous correspondents, the species of insects which feed upon the Tobacco plant—pointing out their station and name in the *Syst. Nat. Lin.* the peculiarities of their natural history; the effects they produce upon other animals when eaten or applied to the skin, if any such are known; the common or popular appellations by which they are distinguished; whether they are considered as poisonous; if their fluids manifest the properties of the plant upon which they feed, or whether the acrid principle of that plant is so changed by digestion and assimilation, as to lose its original properties. If the insects which feed upon Tobacco should be found to acquire the properties of that plant, whether these properties may not have undergone a change in some particular which may render their acrid and soporific principles more valuable medicines, cannot be determined without an experimental inquiry. There are analogies in insect life, and in the other orders of the animal kingdom, which make it probable that the flies or larvæ which feed on

 the

In the general attention which Tobacco has excited, it was not possible that it could escape the investigation of the chemist. At different periods, and under many varied circumstances, attempts have been made to detect and separate the principle that gives energy and activity to this vegetable: to ascertain whether in the same substance was combined its emetic, cathartic, diuretic, and narcotic qualities; whether those qualities might be separated and singly employed; and what proportion they held with the inert earthy or herbaceous bodies, which possibly give form and stability to the plant. The facts respecting these particulars, as ascertained by chemical analysis, have been so governed or distorted by prevailing theories, that some plain and tangible truth which might convey conviction to the mind, is sought for in vain. With one the active principle has been an undefined volatile spirit, escaping on the application of heat, and leaving behind an inert mass. With another an acid phlegm*, a gross sulphur, or a specific salt, in some way afforded the medicinal or deleterious material. Or the active constituent is again believed to be an essential oil obtained by distillation, and which is so virulent, that small animals are almost instantly destroyed when wounded by a needle dipped into it.† The French Chemists, who have laboured exceedingly on the analysis of every substance, have not overlooked Tobacco. The last of them who have attempted to separate the component parts of this plant, and to seize on the substance in which its virtues are believed more especially to be confined, is M. Vauquelin, who has given in the *Annales de Chimie*, an analysis of the *Nicotiana Tabacum latifolia et angustifolia.* (Tabac a larges feuilles.) The result of M. Vauquelin's experiments shewed that the juice of the *Nicotiana latifolium* contained :—

---

the genus *Nicotiana*, may retain the properties of the species of that genus, or have their juices changed by the process of animalization into some other active substance.

* "Such as are more vexed with rheumatic, or erratic pains, by moderate drinking, should smoak Tobacco, for its *acid or sulphur embarasses those acrid particles, whose points are so ready to pierce into, and fix in the small vessels of the periosteum, and excite intolerable pains.*" Discourses on Tea, Tobacco, &c. by Dr. Thomas Short. 8vo. Lond. 1750.—In another place, this writer (who was, notwithstanding these wanderings into hypothesis, a man of observation and laborious inquiry,) says, smoking is serviceable, on account of the salts of Tobacco breaking and dividing the sizy cohesions of the blood.

† In a Memoire sur le Tabac, &c. the active material is said to be a gum, perfectly soluble in water, but not in alkohol; and that with this gum

1. *Une grande quantité de matiere animale de nature albumineuse.*

2. *Du malate de chaux avec exces d'acide.*

3. *De l'acide acetique.*

4. *Du nitrate et du muriate de potasse en quantité notable.*

5. *Une matiere rouge soluble dans l'alcool et dans l'eau, qui se boursoufle considerablement au feu, et dont je ne connois pas bien la nature.*

6. *Du muriate d'ammoniaque.*

7. *Enfin un principe acre,* volatil, sans couleur, soluble dans l'eau et dans l'alcool,* et qui paroit être different de tous ceux qu'on connoit dans le regne vegetal. *C'est ce principe qui donne au tabac préparé, le caractere particulier qui le fait facilement distinguer de toute autre preparation vegetale.*†

---

gum a considerable portion of muriated kali is intimately mixed. Vid. *Journal de Physique*, 1791.

* Le principe âcre dont il s'agit, a peu d'odeur quand il est dissous dans l'eau, ce qui annonce qu'il n'est pas très-volatil ; il paroit très-difficile à détruire, car, mêlé avec une assez grande quantité d'acide muriatique oxigéné, il conserve encore toute son âcreté après que celui-ci s'est évaporé spontanément.

La saveur âcre et la volatilité tout-à-fait particulières de ce corps, semblent indiquer que c'est un principe qui appartient exclusivement au genre nicotiane, et qui par cela même est nouveau, puisque les chimistes qui ont donné l'analyse de cette plante, n'en ont point parlé, au moins à notre connoissance.

† *M. Vauquelin* conjectured that there existed in the species of the genus Nicotiana, a principle very different from any found in other plants ; or if it were not so, Tobacco (that is the prepared Tobacco of Commerce) might be made from a variety of plants ; but by the want of this all endeavours of that sort have been frustrated. Under this impression, assisted by M. Roubiquet, jun. and M. Warden, M. Vauquelin undertook this Analysis. It may not be unacceptable to those who wish to repeat or to pursue the Analysis of M. Vauquelin, to have, in detail, the process of that ingenious chemist, as inserted in the Annales de Chemie, July 1809.

*Procedé.*

" Après avoir broyé les feuilles du nicotiana latifolia, dans un mortier de marbre, on les a enveloppées dans un linge serré, et soumises à l'action de la presse. Pour séparer tout ce que ces feuilles pouvoient contenir de soluble, on a répété trois fois l'opération ci-dessus, en ajoutant une petite quantité d'eau.

Quoique le linge dans lequel on avoit pressé se végétal fût assez serré, le suc contenoit une grande quantité de matière verte en suspension, qu'on

The medical history of Tobacco may be divided into two distinct branches. One will comprehend its employment as

---

qu'on a séparée par la filtration à travers un papier joseph: cette matière verte, restée sur le filtre, a été lavée et mise à part; nous en reparlerons dans la suite.

*Examen du suc filtré.*

1°. Ce suc rougit fortement le papier de tournesol, preuve qu'il contient un acide libre;

2°. L'oxalate d'ammoniaque, par le précipité abondant qu'il y forme, démontre la présence de la chaux; et conséquemment d'un sel calcare quelconque;

3°. Le nitrate d'argent détermine dans le suc de tabac, un précipité abondant qui n'est pas entièrement dissous par l'acide nitrique, d'où l'on peut conclure qu'il est en partie formé par un muriate;

4°. L'infusion de noix de galle, les acides minéraux annoncent, par les précipités bruns assez volumineux qu'ils y occasionnent, l'existence d'une matière animale et particulièrement de l'albumine;

5°. La chaleur élevée à quatre-vingts degrés de l'échelle de Réaumur, en y déterminant une coagulation abondante, confirme ce que les acides et la noix de galle ont anoncé;

6°. L'acétate de plomb y forme un dépôt grisâtre extrêmement abondant, qui se dissout en grande partie dans le vinaigre distillé.

L'effet de l'acétate de plomb dans ce suc, nous ayant fait soupçonner la presence de l'acide malique, nous avons précipité, au moyen ce l'acétate de plomb, une assez grande quantité de cette liqueur coagulée par la chaleur; nous avons fait passer ensuite sur ce précipité lavé et délayé dans l'eau, un courant de gaz hydrogène sulfuré jusqu'à ce qu'il y en ait eu un léger excès.

L'objet de cette opération a été de réduire le plomb en sulfure, et par ce moyen de le séparer de la matière à laquelle il étoit uni. Pour faciliter la précipitation du plomb sulfuré, nous avons chauffé et filtré la liqueur.

Cette liqueur, ainsi filtrée, a été évaporée avec ménagement jusqu'en consistance de sirop; en cet état elle avoit une saveur très-acide, rougissoit fortement l'infusion de tournesol, formoit avec l'alcool et l'ammoniaque, des dépôts abondans qui, en annonçant la présence d'une matière animale, prouvoient qu'une portion de cette substance avoit été entraînée par le plomb dans sa précipitation.

Espérant que l'acide contenu dans cette liqueur épaissie, seroit soluble dans l'esprit-de-vin, et qu'on pourroit par ce moyen le séparer de la matière qu'il tenoit en dissolution, nous l'avons traité à chaud par cet agent à quarante degrés. En effet, aussitôt que le mélange de ces matières a eu lieu, il s'est produit une coagulation abondante, l'alcool s'est coloré, d'abord en jaunâtre, puis en rouge-brun, et est devenu.

La portion de matière non dissoute par l'esprit-de-vin, étoit blanchâtre, se dissolvoit en partie dans l'eau, et sa dissolution précipitoit par l'acétate de plomb comme l'acide lui-même.

L'oxalate d'ammoniaque y occasionnoit un précipité, et cette substance mise a luxury, and its use as a prophylactic; the other will apply to its direct exhibition as a remedy for existing disease.

---

mise sur les charbons ardens, laissoit un residu de carbonate de chaux; enfin nous avons reconnu que cette substance étoit formée en grande partie de malate de chaux qui retenoit quelques portions de matière végéto-animale.

La majeure partie de cette substance végéto-animale que nous avions cherché à séparer au moyen de l'alcool, s'y étant dissoute à la faveur de l'acide, nous avons saturé ce dernier par l'ammoniaque qui y a formé un dépôt floconneux considérable, dont les propriétés étoient entièrement semblables à celles des matières animales. Malgré cette saturation, la noix de galle produisoit encore dans la liqueur un précipité très-sensible; de là l'on voit qu'il existe entre l'acide et ce principe animalisé, une très-grande affinité.

Cet acide purifié le mieux possible, nous a présenté tous les caractères de l'acide malique, c'est-à-dire, qu'il donnoit beaucoup de consistance à l'eau par l'évaporation, ne cristallisoit point, précipitoit l'acétate de plomb en une substance soluble dans le vinaigre distillé, se boursoufloit au feu en répandant une odeur de caramel, et se convertissoit en acide oxalique par le moyen de l'acide nitrique.

Ainsi, l'acétate de plomb avoit tout à-la-fois précipité l'acide malique, beaucoup de matière végéto-animale colorée, et un peu de malate de chaux. Ce dernier paroît avoir été entrainé en combinaison avec le malate de plomb, et avoir été redissous par l'acide malique, a mesure qu'il a été mis a nu par l'hydrogène sulfuré.

Dans plusieurs expériences où nous avions aussi précipité du suc de tabac avec de l'acétate de plomb en excés, nous avons de même retrouvé du malate de chaux dans l'acide malique.

Il existe donc dans la nicotiane une grande quantité de malate de chaux que l'on peut obtenir directement en faisant évaporer aux deux tiers le suc de cette plante.

Une fois la nature de l'acide bien constatée, nous avons repris le suc de nicotiana, dans lequel nous avions versé de l'acétate de plomb en excès, pour le traiter aussi par l'hydrogène sulfuré; nous avons obtenu un liquide bien transparent, de couleur citrine, qui conservoit la même odeur et toute l'âcreté du suc entier. Soupçonnant que cette saveur dépendoit de la présence d'une huile volatile, nous avons distillé la liqueur, et nous avons obtenu un produit qui avoit une légère odeur herbacée et peu de saveur.

La portion concentrée qui restoit dans la cornue, mêlée avec un peu de potasse ou d'ammoniaque, exhaloit une odeur vive et tellement pénétrante, qu'en la respirant un peu fortement, elle faissoit éternuer et couler les larmes; nous répétâmes cette expérience en employant la potasse sur une quantité plus considérable de matière, et nous distillâmes après avoir étendu d'un peu d'eau. Le nouveau produit que nous obtînmes dans cette deuxieme opération, avoit la même odeur que la fumée de tabac, étoit extrêmement âcre, et produisoit une sensation semblable a celle qu'on éprouve lorsque la poudre de tabac, respirée trop fortement tombe dans la gorge.

Comme

Of its general employment as a luxury, and of the evils it produced, or was supposed to have produced, on the morals and in the affairs of mankind, enough has been said in the former part of these Remarks.

Its power to preserve from disease, and to prolong life, has been the fond theme of many writers, who persuaded themselves that by its constant use the extreme period of longevity might be attained, and that the interval would be filled with jollity, uninterrupted health, and perfect happiness. A Dutch Physician,* in a work published in 1690, was so convinced of its power to effect those desirable objects, that he concludes, in the last chapter (the 20th) of his elaborate volume, that every man and woman should be encouraged or compelled to smoke, that they may pass a life of gaiety and unvarying health.† His work begins with assert-

---

Comme ce produit étoit alcalin, nous avons soupçonné que ce principe, quel qu'il fut, ne se volatilisoit qu'a la faveur de l'ammoniaque, provenant de la décomposition d'un sel ammoniacal contenu dans le tabac, puisque quand la liqueur étoit avec excès d'acide, nous n'obtenions point le même résultat. Cependant dans une opération semblable, fait a la vérité sur un tabac sec, nous avons obtenu un produit dont l'odeur et la saveur étoient pour le moins aussi prononcées, quoique la liqueur d'ou il provenoit contînt un acide libre. Au reste, par la distillation, nous ne sommes jamais parvenus a isoler parfaitement cette substance acre, et même la plus grande partie restoit dans la cornue : il paroit d'apres cela que l'acide malique diminue la volatilité de ce principe acre.

Pour tâcher d'obtenir séparement ce principe, nous avons évaporé à une trés-douce chaleur, la liqueur qui le contenoit, et nous l'avons traité par l'alcool à quarante degrés qui, en effet, l'a séparé des autres matieres : en faisant ensuite évaporer l'alcool, nous avons remarqué a la surface du liquide, quelques traces d'huile brune, et la porcion qui passoit a la distillation, acquéroit d'autant plus d'âcreté, que l'opération arrivoit plus pres de sa fin. Cette huile presque solide répandoit, lorsqu'on la mettoit sur un charbon allumé, une fumée épaisse, et une si forte odeur de tabac, qu'elle en étoit insupportable.

Cette dissolution alcoolique a fourni, en refroidissant, du nitrate de potasse en quantité notable.

* Joannes Ignatius Worp Beintema Van Peima, M. D. *Tabacologia, ofte Korte Verhan delenge over de Tabak, Defselvsdeugd, gebruyk ende kennisse: Waar door aangeweesen word't een wegh om lang, vroolyk, ende gesond te leeven.* 12mo. Gravenhage. 1690. pp. 175. With a frontispiece of men and women smoking, worthy of Hogarth.

† Met aanmoedigeng van Tabak te rooken aan alle mans, ende vrouwen, met een bewijs, dat se verpligt zyn te rooken, so se blyde, vrolyk, ende wel leevende wille zyn.

So highly was Tobacco esteemed at this period, that *Johannes Kiesenga,* who presented complimentary verses to *Beintema* on his publication, contends that the discovery of this plant alone has placed the science and knowledge of the moderns above that of the ancients.

ing, " *Het rooken is een van de voornaamste middelen, om gesondheyd, ende leeven lang te behouden.*" That smoking is one of the principal means to preserve health and to prolong life.

The prophylactic properties of Tobacco were once, however, believed to be more specifically determined.* Diemer-

Indien de gryse Eeuw uyt haar gefronste kuilen
Ons leev tijds wijsheid, en dien drom van letter—zuilen,
Aansag ; gewisselyk sy zeid geheel versuft :
Nu is't de vyvde Eeuw van 't Goddelyk vernuft.
Neerland alleene geeft meer ligkt, als in myn dagen
En Greik-en Romerland met al haar Wijsen saagen.
Swijg vry Aaloude tijd ! en trek 't gerimpold hoofd
In't swarte grav, dees Eeuw heeft u van konst beroofd.
Ganz bar sou dit geluyt ter schorre keel uytstygen,
(Niet met maarteegen wil) indiense niet moest swygen.
Alleen Genees-kunst die in onse daagen praald
Toond, dat de wetensghap nu word voor't ligt gehaald.
In alles vind men kraght om swakke wear te styven,
Uit hout, uit been, selvssteen daarkan men kragt uit dryven.
Sghoon dat de oudheid hier in minst geen deugd in keurt,
Word nogtans in ons tijd hier groote kragt bespeurt.
Ons kruyd, wiens naam dat selvs de oudheid nimmer kende,
Roemt sig een troost te zyn, een hulp voor veel elende.
Prijswaardig is by dan, die ons dat kruyd verklaart,
Besghermt, en al sijn kraght, en dengden openbaart,
En toont met hoeveel heyl dit heerlyk kruid kan werken
In 'smensghen lighaam, hoe't de geest selv kanversterken,
Naadien het sinnelyk door rook met rook omswiert,
Terwijl de geest haar selv tot hooger dingen stiert,
En soekt naa 't vaste goed, 't welk niemand kan bevatten
Met sinnelyk gewoel dat nooyt so hoogh kan spatten.
Aaloude Eeuwen swijgt dan vry maar van u lof,
Van welk de wereld waagt, gy sghreeft nooit van een stof
Als deese is : de kunst godin woont nu op Aarde,
Nu segt se 't geen se u nog nimmer openbaarde.
Prat dan o Haaven-stad vry op u BEINTEMA,
Eer hem, die evenschreets Heer *Bontekoe* gaat na ;
Juight vry om dat hy sig, en ook U met zyn schriften
Met onverwelkbaar lof dees eerezuil gaat stifren.
Apollos voesterkind ; was *Dokkums* Voesterling.

* Though Diemerbrook, who was extremely conversant with the subject, had an high confidence in the prophylactic virtues of Tobacco in Pestis, and others also recommended it with some earnestness ; yet there is much reason to conclude it has no property that renders it valuable in that view. Dr. Russel, who had the best means of making observations on this subject, says, that those who employed Tobacco as a preservative brook asserted that it preserved from the contagion of the Plague; and that all the inhabitants of houses both in London and Nimeguen, where tobacco was sold, escaped infection, while those in the adjoining ones received the disease. But this property was denied by Rivinus and others, who affirm, that many great smokers died in the Plague of Leipsic. Dr. Thomas Short asserts its prophylactic property, with regard to contagious and epidemic distempers: and though his reasoning on its *modus operandi* cannot be admitted, yet the facts he mentions are, if fully ascertained, of great value.* A more exact acquaintance with the laws of

---

preservative (at Aleppo) were not less liable than others to be infected. The custom of smoking is universal among both men and women of all ranks at Aleppo, and Dr. Russel supposed that the habit might deduct from its prophylactic property, but corrects himself by remarking, that those who use it as a preservative must in some degree be accustomed to it, otherwise the violence of its operation might prove hurtful. But as the Tobacco commonly used in Syria is much milder than the American, this remark may lose some of its force. Dr. Mead hesitatingly recommends smoking as a prophylactic in Pestis. Further observations on this property in Tobacco may be seen in *Orræi descriptio Pestis.* Primrose, (*De vulgi erroribus in Medicina*, 16mo. Amstelod, 1639.) has some curious reasoning on the prophylactic property of Tobacco in the Plague. His opinions cannot be considered as vulgar errors; but the mistakes of the learned are of more importance than those of the unlearned. Adamus Hahn (*Tabacologia sive de Tabaco dissertatio.* 4to. Jenæ,) thought its prophylactic property of sufficient importance to make one of the queries in his 7th chapter; which especially inquires if smoking Tobacco is serviceable against the Plague? See De Merten's Obs. Med. de Febr. putr.—Chenot Tract. de Peste. Sir William Temple thought it not only cured diseases of the eyes, but preserved the sight. His words are, " For rheums in the eyes and the head, I take a leaf of Tobacco put into the nostrils for an hour each morning, to be a specific medicine. An old prince Maurice of Nassau told me, he had by this preserved his eyes to so great an age, after the danger of losing them at thirty years old: and I have ever since used it with the same success, after great reasons, near that age, to apprehend the loss or decay of mine." Essays, 8vo. vol. iii. 297. This is cited rather as a specimen of opinions which once prevailed, than as a practical precept.

* " In time of pestilence, or any other contagious or epidemic distemper, smoking is profitable, both as the pungency of the smoke accelerates the blood's motion, and prevents the alteration of our mass of blood, by the attraction of the contagious effluvia drawn in with our breath, and swallowed with our spittle, but especially as its much sulphur embarrasses and sheaths those effluvia; *hence all the Tobacconists' houses and shops in London barred the entrance of the spreading contagion in* 1665 *and* 66; even the fumes of the Tobacco, by stirring it up and handling nature, profound chemical investigations, and an experimental knowledge of the operations of animal life, have taught modern physiologists to reject all preservatives from contagion, which do not chemically neutralize or destroy the contaminating material. Fumigations, and odoriferous substances, camphor, ruta, &c. are not only useless, but possibly increase the danger. The organ of smell has been given to animals, not only for the purpose of discriminating food, but as a sentinel to give warning of the approach of danger.* When enjoying the full exercise of its natural

---

handling it, had destroyed the venom of the malignant effluvia, before they were sucked in with the people's breath." Discourses on Tea, Tobacco, &c.

* The abuse of the organs of sense, in a highly artificial state of society, have frequently so injured their properties, that civilized man has hardly an idea of their acuteness in a state of nature. Savage tribes in the American forests know if a man has passed the preceding day, by smelling to the leaves or herbage on which he has trampled. A dog scents game at considerable distances; and, if the fact were not confirmed by daily experience, it would hardly gain credit, that he can trace the odour of his master's foot through all the winding streets of a populous city. A very ingenious collector of the facts in natural history, speaking of the *sense of smell*, says, "all bodies in nature, whether solid or fluid, whether animated or inanimated, continually send forth to the air certain effluvia or emanations. These effluvia float in the atmosphere, and act upon the olfactory nerves of different animals, and sometimes of different individuals of the same species, in such a manner as to produce very different sensations. Brute animals select their food chiefly by employing the sense of smelling, and it seldom deceives them. In the selection of food, men are greatly assisted, even in the most luxurious state of society, by the sense of smelling. Substances of a putrid smell, as equally offensive to the olfactory nerves and to the constitution, are avoided with abhorrence and rejected as noxious. The more acute discernment of brutes in the exercise of this sense, is entirely owing to their freedom, and to their using natural productions alone. But men in society, by the arts of cookery, by the unnatural assemblage of numerous ingredients in one dish, *blunt, corrupt, and deceive their sense of smell.* Were they in the same natural condition as the brute, they would be enabled by the olfactory organs to distinguish, with equal certainty, noxious from salutary food. Domestic animals are nearly in the same condition with luxurious men. A pampered dog snuffs and rejects many kinds of food, which, in a natural state, he would devour with eagerness. But assistance in the choice of food is not the only advantage that men and other animals derive from the sense of smelling. The subtilty of the emanations that are sent forth from animal, vegetable, and mineral substances, when exposed to the air, is so extreme, that no eye can perceive them. These effluvia, or volatile particles, are diffused through the air, and are recognized by the organ of smell. To powers, the olfactory apparatus will sometimes, perhaps frequently, give intelligence of being within the sphere of the infecting atmosphere. How absurd, then, is it to confuse this sentinel by a variety of powerful odours, which certainly do not correct or destroy the infecting gas, or whatever it is, but merely render the nerves of the organ of smell insensible to it!

The confidence with which Tobacco was used as a prophylactic, was certainly equalled by the opinion that it produced singular changes in some of the organs. Many writers, and some of great credit, believed, that the brain of immoderate smokers became incrusted with a fuliginous matter similar to soot in a chimney.* It is more certain, or at least more con-

---

give some idea of the minuteness of these particles, and of the amazing sensibility of the nostrils of animals, it is only necessary to state, that the odour of musk has been known to fill a large space for years without losing any perceptible part of its weight. Thus the air we breathe is perpetually impregnated with an infinity of different particles which stimulate the olfactory nerves, and give rise to the sensation of smell. When our senses are not vitiated by unnatural habits, they not only convey to us exquisite pleasure, *but are the faithful monitors of danger.*" It is probable that all the pestiferous gases, or the materials of contagion, whatever they are, have connected with them a mauvais odeur that will induce to a removal from their sphere of action. I know but of one exception. Some of the most noxious vapours in mines are also attended with a delightful smell, resembling the pea-blossom. These vapours generally come in the summer, and oblige the miners to quit their work, to whom they would otherwise soon prove fatal. *(Phil Trans.)* In that exquisite poem, the Mine, the author has touched this subject with great taste. He adopts the Rosycrusian fancy of Gnomes, spirits which inhabit the earth, and who by their power form the ores of metals, and all the wonders met with in the inmost recesses of the globe.

"Wheresoe'er our footsteps turn,
Rubies blush, and diamonds burn;
Every grot and silver cave
Streams of milk and amber lave;
*And our bow'rs such perfumes give*
*As mortals cannot taste and live.*"
SONG OF THE GNOMES.

* ADAMUS HAHN, before cited, in a series of questions on the properties and uses of Tobacco, has one "*An fumus Tabaci crustus nigrans in cerebro gignat?*" The response to this question gives an epitome of opinions which once prevailed.

"Quilibet pro sua sententia confirmanda experientiam adducit. Ita ex affirmantium ordine se sistit. *P. Pavius Lugdun. Anatomicus*, qui in juvene cultro anatomico subjecto tales se invenisse crustas gloriatur; sonant to observation, that the brain, though not encrusted with Tobacco soot, has often suffered by the constant use of

---

tur; fuit verò fumifugus celeberrimus. *Falckenburgius, qui in Epist. ad Neandam* profitetur se easdem observasse. Rationes sunt, 1. quod tabacum in se habeat unctuosam, oleosam, viscosam materiam, quæ successu temporis viscidæ & tenaci cerebri substantiæ poterit adhærere, atque talem concretionem facere. 2. spontanea ad superiora fumi ascensio & pororum cerebri ad recipiendum aptitudo adsit. A negantium parte stant *J. Dan. Horst. in manud ad med. C. Magn. l.* c. *p. 111.* qui opponunt illis observationem *Gvil. van der Meer*, fatentis, se in pluribus fumifugis dissectis tale quid invenire non potuisse, imò se in fure καπνοφίλῳ celeberrimo, ita ut in ipsa quoque hora mortis fumum hunc avidius sumserit, tale quid cerebro dissecto, observare nequivisse. Ita in virili cadavere, (quod dum erat in vivis, avidius fumum hunc hauriebat) peritæ ante biennium *Nobiliss. atque Excell. Dn. D. Præsidis* sectioni subjecto non licuit quidquam talium crustarum deprehendere. Hoc tamen in ipso notabatur, quod sinistra cerebri substantia sese totam putredine corruptam, cum colore ad cæruleum vergente, exhiberet. Num verò hæc corruptio a nimio fumi hujus usu ortum traxerit, (cum dextrum latus adhuc salvum, hic verò ad utrumque; æque possit ferri) aliis judicandum offerimus. *Hoferus* alio exemplo adducto, hoc itidem negat, similiter *Primir. l. 4. c. 34.*, rationes habent 1. quod si fuligo talis fieret sensibilis, non possit aliter fieri, quin inducat gravissima symptomata. 2. quod aër non feratur ad cerebrum, de quo vid. *CL Schneider. l. c. Higmor. disquisit. anatom. p. 222.*

Pro affirmantium parte faciunt illa, quæ habet *Hoffman. l. c.* Audivi, *inquit*, a militibus in Belgio versatis, vidisse se dissecta capita eorum, quibus patera ab anatomicis dicta, tota interius esset nigra, licet a carnifice interiissent. Audivi, *pergit*, a patritio Norico, qui in superiori bello Bohemico vidit omnes, qui in conflictu cum hostibus interierint, Anglos, habere talia capita. Pro his etiam facere posset aëris in cerebrum ingressus, quem concedit *Excell. D. D. Rolfinc. in dissert. anat. p.* 711, & 569, attractio aeris, *inquit*, fit per ossis cribrosi foramina, & non solùm aër cerebri meningem ambit, sed & ventriculos ipsos ingreditur. Quo cum facit etiam *Nobiliss. Dn. D. Schenckius in Schola Part. p.* 42. Cum aere ergò, dixerit aliquis, junctus fumus hic, utpote summe penetrans, atque ad cerebrum delatus, unctuositate, lentore ac pinguedine sua tales generare posset crustas, si non ita palpabiles, tamen visibiles.

Nos interim hæc decidenda doctioribus relinquimus, contenti jam opinionum quarundam recensionibus, circa quas ut quilibet suo sensu abundet, æquo fermius animo, addendo saltim ex *C. Lang. in Misc. C.* exempla & observationes in arte medica quidem plurimi esse momenti, sed illa tamen cum ratione conjungenda."

The excellent and laborious Mongagni did not think this subject quite undeserving his notice, and shews, in his great work, "*de Causis et Sedibus Morborum*," that when this fuliginous matter was detected by dissection, it either arose from some other cause, or the anatomist was imposed on by some legerdemain trick.

 this this narcotic, (we shall see hereafter that a very contrary opinion has prevailed); and that when used in the form of snuff the perfection of the organ of smell has always been injured. My own observation has led to a conclusion, that the constant and profuse employment of this herb has been injurious to the brain and nervous system. In twenty-five years actual practice, a great number of cases of *Paralysis* have come under my notice; in all, or in the far greater part of these, the men were smokers, and the women snuff-takers.*

*(To be concluded in the next Number.)*

---

* It is not to be doubted that the immoderate use of Tobacco has, in some idosyncracies, produced alarming effects on the most efficient organs of the animal machine. "I have observed," says Cullen, "several instances of great snuff-takers being affected in the same manner as persons are from long continued use of other narcotics, such as wine and opium; that is, *by loss of memory, by fatuity, and the other symptoms of the weakened or senile state of the nervous system, induced before the usual period.* I have found also," adds this ingenious physician, "that excessive snuff-taking produces all the symptoms of dyspepsia, particularly pains of the stomach, occurring every day." Almost two hundred years ago, Dr. Venner, in the quaint but forcible language of that time, objected to the use of Tobacco. "I will summarily rehearse," says the author of the Via Recta, "the hurts that Tobacco inferreth, if it be used contrary to the order and way I have set down. It drieth the brain, dimmeth the sight, vitiateth the smell, hurteth the stomach, destroyeth the concoction, disturbeth the humours and spirits, corrupteth the breath, induceth a trembling of the limbs, exsiccateth the winde pipe, lungs, and liver, annoyeth the milt, scorcheth the heart, and causeth the blood to be adusted. Moreover it eliquateth the pinguic substance of the kidnies, and absumeth the geniture. In a word, it overthroweth the spirits, perverteth the understanding, and confoundeth the senses with a sudden astonishment and stupiditie of the whole body. All which hurts I affirme, that the immoderate and intempestive use of Tobacco doth affect, both by reason of its temperament (hot and dry in the third degree); but especially through the propertie of its substance (deleteriall or venemous): Wherefore the use of it is only tolerable by way of physick, not for pleasure or an idle custome. To conclude, therefore, I wish them that desire to have *mentem sanam in corpore sano*, altogether to abandon *insanum præposterumque Tabacci usum.*" Via Recta ad Vitam Longam, 4to. Lond. 1638. p. 363. The destroying spirit of man taught the wandering savages of America to envenom their arrows with a poison prepared with this plant. The black pigment which collects in long-used tobacco-pipes, is a strong poison to some animals. Barrow (Travels in Africa) relates that he saw a snake poisoned with it. The effect was instantaneous as the electric shock. I remember to have seen the common English snake thus instantaneously destroyed. Barrow asserts, that the snake he saw thus killed

*To the Editors of the Medical and Physical Journal.*

GENTLEMEN, killed became immediately hard and rigid, as if dried in the sun. The Hottentots consider this substance, which they call oil of tobacco, as the most deadly of poisonous substances; but it is never applied to the points of their arrows, because it is too volatile to retain its deleterious quality. One Manwaringe, a chemical doctor, or a trading chemist, in 1666, brought a severe charge against tobacco. He accused it of producing scurvy, and wrote a duodecimo pamphlet to prove his accusation.

